# Recurrence of thyroglossal cyst: on the tongue!

**DOI:** 10.11604/pamj.2021.38.329.23259

**Published:** 2021-04-06

**Authors:** Vanesa Villamil, Nery Alfredo Méndez Aguirre

**Affiliations:** 1 Pediatric Surgery Service, Sant Joan de Déu Children’s Hospital, Barcelona, Spain,; 2 Pediatric Surgery Service, University Clinical Hospital Virgen de la Arrixaca, Murcia, Spain

**Keywords:** Thyroglossal duct cyst, tongue disease, recurrence, children

## Image in medicine

Thyroglossal duct cysts (TDC) are the most common congenital cervical abnormality. On the other hand, lingual TDC are rare, comprising between 0.6% -3% of all TDC. We present the case of a patient with recurrence of the TDC, at the base of the tongue. Five-year-old patient with TDC was admitted on a scheduled basis to excise it. Exeresis of the cyst was performed using the Sistrunk technique. Postoperative elapsed without incidents, being discharge on the 3^rd^ postoperative day. Three years after surgery, the patient returned for dysphagia and the appearance of a tumour on the base of the tongue (A). Cervical ultrasound was performed, showing a suprahyoid cystic structure, adjacent to the posterior third of the tongue, dependent on the blind foramen, compatible with lingual TDC (B). The patient underwent an intraoral approach, allowing complete removal of the cyst. Pathological anatomy confirmed the suspicion of TDC. TDC form anywhere from the blind foramen of the tongue to the base of the neck. Sixty one percent of the cysts are located between the thyroid gland and the hyoid bone and 1-2% are intralingual. Cases of transoral exeresis and using the Sistrunk technique are reported. We performed the first one, since the patient had already undergone cervical surgery. The exploration of the oral cavity in these patients is essential, since, as we have seen and learned in this case, there may be a recurrence or extension of the cyst to the blind foramen of the tongue.

**Figure 1:**
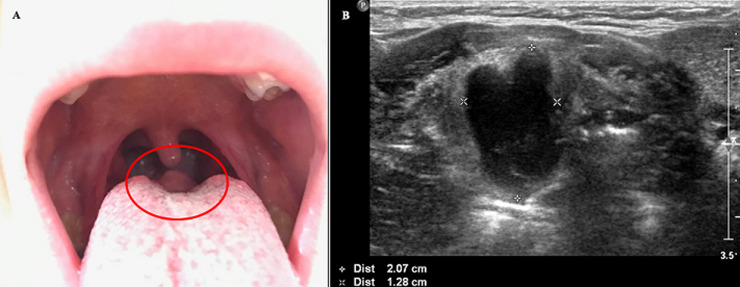
A) tumour in the posterior base of the tongue, compatible with lingual TDC; B) ultrasonographic appearance of the lesion, adjacent to the blind foramen of the tongue

